# Improving Utstein accuracy: concordance of bystander CPR reporting by paramedic documentation vs. telecommunicator audio review

**DOI:** 10.1016/j.resplu.2025.101122

**Published:** 2025-10-10

**Authors:** Helen N. Palatinus, Ashlynn A. Felker, Tate Colton, Graham Brant-Zawadzki, Scott T. Youngquist

**Affiliations:** a The Department of Emergency Medicine, Spencer F. Eccles School of Medicine at the University of Utah, Salt Lake City, UT, United States; b Unified Fire Authority, Salt Lake City, UT, United States; c Salt Lake City Fire Department and 911 Dispatch, Salt Lake City, UT, United States

**Keywords:** Cardiopulmonary resuscitation, Bystander CPR, Emergency medical services, Telecommunicator, Patient care records

## Abstract

**Background:**

Bystander cardiopulmonary resuscitation improves outcomes for out-of-hospital cardiac arrest. While registries typically rely on patient care reports for bystander CPR documentation, the accuracy of reporting is unknown. This study aimed to determine the agreement in bystander CPR reporting between patient care reports and audio review of public safety answering point emergency calls.

**Methods:**

In this retrospective study, we analyzed paired patient care reports and dispatch audio files for bystander CPR documentation. Cases dispatched by a secondary public safety answering point, emergency medical services-witnessed arrests, or those with missing data were excluded. We compared documented rates of bystander CPR from each source, calculated inter-rater agreement, and identified factors associated with reporting.

**Results:**

The concurrence in reporting was 72.2 %, with a moderate inter-rater agreement between the two methods (κ = 0.402, 95 % CI 0.341–0.463). Audio review documented higher bystander CPR than patient care reports (74.8 % vs. 57.0 %). An initial shockable rhythm (adjusted odds ratio (aOR) 1.68, 95 % CI 1.18–2.40 in patient care reports; aOR 1.57, 95 % CI 1.04–2.36 in audio files) and advanced life support unit first on scene (aOR 1.55, 95 % CI 1.15–2.07 in patient care reports; aOR 1.51, 95 % CI 1.08–2.11 in audio files) were associated with higher documentation.

**Conclusion:**

We found moderate agreement in bystander CPR documentation between audio files and patient care reports, with a higher incidence of bystander CPR recorded in dispatch audio. These findings suggest inconsistencies in bystander CPR documentation across the emergency response system, highlighting the need for standardized reporting to ensure accurate data collection.

## Introduction

Bystander cardiopulmonary resuscitation is a critical determinant of survival in out-of-hospital cardiac arrest (OHCA), approximately doubling patient survival.^1^ Consequently, there exist numerous emergency medical services (EMS) and public health initiatives aimed at increasing bystander CPR rates with the hope of improving outcomes.^2^, ^3^ Accurate data collection on bystander CPR is essential for evaluating the effectiveness of public health interventions and understanding its impact on OHCA outcomes. However, current data collection practices exhibit considerable heterogeneity. Registries such as the Cardiac Arrest Registry to Enhance Survival (CARES) acquire bystander CPR information almost exclusively from EMS patient care reports.^4^ Unfortunately, EMS patient care reports may contain missing, inaccurate, or unreliable data.^5^, ^6^, ^7^ For example, bystander CPR may be stopped prematurely before EMS arrives on scene and, without directly questioning bystanders, EMS may assume that no bystander CPR has been performed if it was not observed directly.^8^ Time constraints, competing priorities, and challenges in recall may also play a role in the incomplete collection of data or documentation.^5^, ^6^ Telecommunicators, conversely, are typically the initial point of contact for cardiac arrest identification and prearrival CPR instructions and thus, “witness” the performance of bystander CPR – at least as much as can be observed by audio only.^3^ This study aimed to assess the agreement between EMS patient care reports and review of 9-1-1 dispatch recordings for the documentation of bystander CPR performance. A secondary outcome of this study was identifying factors associated with bystander CPR documentation by paramedics. We hypothesize that discordance in reporting will exist, with telecommunicators demonstrating a higher rate of bystander CPR documentation. The goal of this investigation is to quantify the difference.

## Methods

### Study design and setting

We performed a retrospective study of prospectively collected data comparing the reporting of bystander cardiopulmonary resuscitation between EMS patient care reports and review of emergency call audio files in all consecutive out-of-hospital cardiac arrests treated by a single EMS agency between August 2016 and February 2025.

The study was reviewed and approved by the Institutional Review Board of the University of Utah (IRB_00138043).

The Salt Lake City Fire Department is an urban two-tiered EMS agency in Salt Lake City, Utah. The department provides prehospital care to a population of 200,000 nighttime residents, which more than doubles during the daytime.^9^ It manages 35,000 annual calls for service and provides care for approximately 130 cardiac arrests annually.^10^ Public Safety Answering Point (PSAP) 9-1-1 calls are managed by dispatch operators who use the Medical Priority Dispatch System (International Academies of Emergency Dispatch, Salt Lake City, UT) to triage and guide pre-arrival care.

Paramedics variably record data into the electronic patient care report on a tablet either on scene and in real-time or post-incident and enter Utstein variables into a required template when the primary impression is cardiac arrest (ESO Suite, ESO Solutions, Austin, Texas until 5/5/2016 and then ImageTrend, ImageTrend Corp, Eagan, Minnesota thereafter).^11^ Paramedics document the provision of bystander CPR through a pull-down menu or enter the data in the free text narrative. Public Safety Answering Point 9-1-1 calls are recorded and archived and, beginning in August 2016, were collected for audio review and telecommunicator feedback by the dispatch medical director (SY). Since December 2011, treating EMTs and paramedics had received CPR feedback and outcomes data from the medical director’s office. Starting in August 2016, telecommunicators were provided with feedback and patient outcomes as well.

The department maintains an active Salt Lake City Fire Department Cardiac Arrest Registry. Trained data abstractors compile data from EMS patient care reports into the Registry according to Utstein Out-of-Hospital Cardiac Arrest Registry Template criteria.^11^ The medical director (SY) reviews all data for accuracy and reviews the audio and a written transcript of the dispatch call before sending feedback and outcomes data to both dispatchers and EMS clinicians. Patient data is recorded from time of call for service to time of death or hospital discharge.

### Participants

The study included paired data from EMS patient care reports and public safety answering point 9-1-1 calls for all consecutive, out-of-hospital cardiac arrests treated by the Salt Lake City Fire Department. To be considered for the database the patient had to have received either a defibrillation from a public access automated external defibrillator (AED) or CPR from EMS personnel. Patients with hanging/strangulation, traumatic causes of arrest, drowning, or exsanguination are excluded from the database. The primary analysis excluded cases with missing 9-1-1 dispatch audio files due to the arrest occurring at the Salt Lake International Airport Public Safety Answering Point, calls taken by a secondary Public Safety Answering Point, or secondary dispatches from police. We also excluded EMS-witnessed arrests and cases with no comment on the provision of bystander CPR in the patient care report.

### Exposure

The exposure was the provision of bystander CPR as reported by paramedics and 9-1-1 dispatch audio review. We defined bystander CPR as CPR provided by individuals who are not a part of an organized emergency response system.^1^

### Outcome

The primary outcome was concordance in documentation on the provision of bystander CPR among dispatch calls and patient care reports. We also assessed the prevalence of bystander CPR administered as reported by dispatch files and patient care reports. We also assessed the association of Utstein variables and EMS personnel level with the documentation of bystander CPR.

### Data collection and analysis

Three reviewers (AF, CT, SY) assessed the dispatch audio files and patient care reports of each OHCA and recorded the presence of reporting of bystander CPR and the prevalence of bystander CPR administered. We collected and managed data in the RedCap electronic data capture application hosted at the University of Utah.^12^, ^13^ The data was exported into a statistical software program (Stata 18, StataCorp, College Station, CO) for analysis. We used Cohen's kappa statistic (GraphPad, GraphPad Software, Boston, MA) to measure concordance of bystander CPR documentation between EMS clinicians and telecommunicators. We defined the level of agreement for the kappa statistic for values <0 as no agreement, 0.00–0.20 as slight agreement, 0.21–0.40 as fair agreement, 0.41–0.60 as moderate agreement, 0.61–0.80 as substantial agreement, and 0.81–1.00 as almost perfect agreement.^14^ We presented continuous variables as means with standard deviations and medians with interquartile range based on data distribution. Comparisons between groups for continuous variables were performed using the *t*-test. We performed comparisons between categorical variables using the Fisher exact or chi-square test, as appropriate. Multivariable logistic regression was used to assess associations between predefined factors and outcomes, including the Utstein core variables (age, sex, witnessed arrest, response interval, initial shockable rhythm, and public arrest location) and the level of the first EMS clinician on scene. Adjusted odds ratios (aOR) were reported with 95 % confidence intervals. A two-tailed p-value <0.05 was considered statistically significant.

## Results

**Fig. 1:**
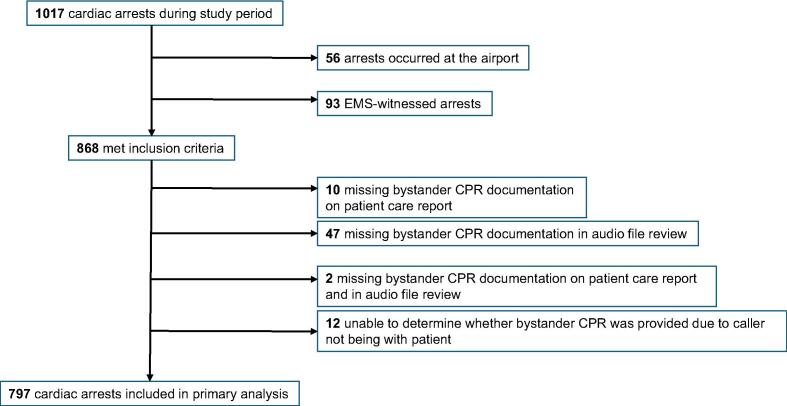
**Selection of study population.** EMS, emergency medical services; CPR, cardiopulmonary resuscitation.

**Fig. 2:**
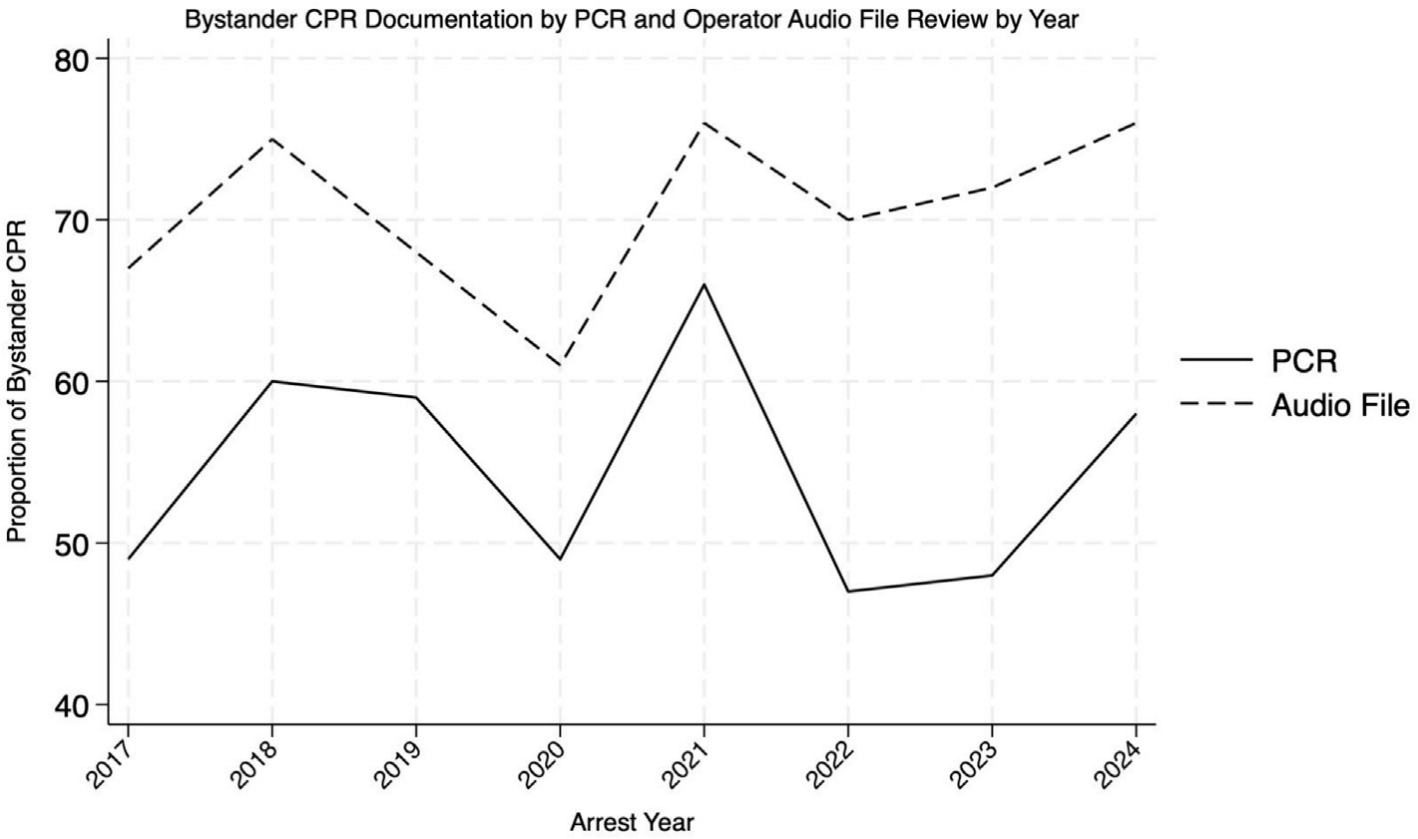
**Proportion of bystander cardiopulmonary resuscitation over time**. CPR, cardiopulmonary resuscitation.

Patient demographic and resuscitation characteristics are as shown in Table 1. Among 1017 OHCAs during the study period, 797 cases were left for analysis after exclusions (Fig. 1). The 9-1-1 audio review resulted in a higher proportion of provision of bystander CPR (74.8 %, 596/797 in audio files, 57.0 %, 454/797 in patient care reports) (Fig. 2). We found moderate inter-rater agreement (kappa 0.402, SE 0.031, 95 % confidence interval 0.341 to 0.463) in documentation of the provision of bystander CPR between prehospital clinicians and telecommunicators. The concurrence in reporting was 72.2 % between the two groups.

**Table 1:** **Study population demographics.** SD, standard deviation; AED, automated external defibrillator; CPR, cardiopulmonary resuscitation; EMT, emergency medical technician; time interval is in minutes.

**Characteristic**	**Study Population** (n = 797)
Mean age (SD)	57.1 (18.4)
Sex	
Male (%)	545 (68.4)
Female (%)	252 (31.6)
Witnessed arrest (%)	395 (49.6)
Public arrest location (%)	209 (26.2)
Bystander AED use (%)	31 (3.9)
Shockable first arresting rhythm (%)	220 (27.6)
Dispatcher CPR instructions (%)	498 (83.0)
First responder	
EMT (%)	340 (42.8)
Paramedic (%)	452 (56.9)
Physician (%)	2 (0.3)
Mean response time (SD)	6.5 (2.2)
Survival to hospital discharge (%)	105 (13.2)
Witnessed arrest (%), n = 395	89 (22.5)
Initial shockable rhythm (%), n = 220	83 (37.7)
Neurologically intact at discharge (%)	104 (13.0)
Witnessed arrest (%), n = 395	88 (22.2)
Initial shockable rhythm (%), n = 220	79 (35.9)

In both paramedic patient care reports and 9-1-1 audio review, there was increased bystander CPR reporting when advanced life support clinicians were the first unit to arrive on the scene (aOR,1.55, 95 % CI in patient care reports, 1.15–2.07, aOR 1.51, 95 % CI, 1.08–2.10 in audio files) and when the patient had an initial shockable rhythm (aOR 1.68, 95 % CI 1.18–2.40 in patient care reports, aOR 1.57, 95 % CI 1.04–2.36 in audio files). The audio file review had a negative association with public location of arrest (aOR 0.67, 95 % CI, 0.46–0.99). There was no association of sex or response interval with bystander CPR documentation (Table 2).

**Table 2:** **Association of demographics and situational factors with patient care report and dispatch audio file reporting of bystander cardiopulmonary resuscitation.** ALS, advanced life support.

**Variable**	**Adjusted Odds Ratio**	
	**PCR Review**	**Audio File Review**
Age	1.00 (0.99–1.01)	0.99 (0.98–1.00)
Sex	0.99 (0.720–1.36)	1.00 (0.693–1.44)
Witnessed arrest	0.73 (0.53–0.99)	1.10 (0.78–1.56)
Response time	0.99 (0.93–1.06)	0.93 (0.86–1.00)
Initial shockable rhythm	1.68 (1.18–2.40)	1.57 (1.04–2.36)
Public location of arrest	1.27 (0.89–1.81)	0.67 (0.46–0.99)
ALS first unit of scene	1.55 (1.150–2.07)	1.51 (1.08–2.11)

## Discussion

In this study, we aimed to assess the agreement in bystander CPR reporting between two sources of prehospital information: EMS clinician documentation and public safety answering point emergency dispatch call review. Our findings reveal a moderate level of agreement (kappa = 0.402) between these two reporting methods, with an overall concordance of 72.2 %. Notably, dispatch audio file review consistently identified a higher rate of bystander CPR administration (74.8 %) compared to EMS clinician documentation (57.0 %). These discrepancies underscore significant inconsistencies in how bystander CPR is captured and recorded across the prehospital care continuum, as has been echoed in a previous study identifying substantial inter-rater variability in OHCA reporting.^15^ Due to a lack of criterion standard, we used concordance as the outcome.

The observed moderate agreement suggests that while EMS documentation and dispatch review often align, a substantial proportion of cases exhibit discordance. This has important implications for the accuracy of cardiac arrest registries, which frequently rely on data from EMS documentation for bystander CPR rates. If dispatch audio, which captures the immediate moments of interaction and instruction, provides a more comprehensive picture of bystander CPR efforts, then current reporting mechanisms may underestimate the true incidence of bystander CPR. The higher rate detected via dispatch review could indicate either under-recognition or under-documentation by EMS clinicians upon arrival, a phenomenon also seen in a prior study on the variability of prehospital bystander CPR reporting. A secondary analysis of a randomized, prospective trial assessed the inter-rater reliability of a consensus panel compared to two reviewers, a patient care report drop-down menu response, and a data sheet completed by an on-scene physician in assessing the provision of bystander CPR. Among the 100 records reviewed, there was moderate agreement between the consensus panel and the PCR drop-down menu (κ = 0.54, SD 0.09). The investigators found substantial agreement between the consensus panel and reviewers (κ = 0.61, SD 0.07; κ = 0.66, SD 0.07) and between the consensus panel and the physician data sheet (κ = 0.68, SD 0.08).^15^

The presence of an initial shockable rhythm and the arrival of advanced life support as the first unit on scene were positively associated with bystander CPR reporting, suggesting that higher levels of initial medical intervention may elicit more consistent documentation.

Data abstraction from multiple sources can yield varying results, particularly in complex, high-stress environments like OHCA. Although direct comparisons between dispatch audio file review and EMS patient care reports are limited, extrapolation from in-house cardiac arrest (IHCA) studies provides some insight. In a study encompassing 52 witnessed IHCA, comparisons of the documentation of interventions were made between a prospective observer and retrospective review of documentation within a “real-time” resuscitator narrator module within the electronic health record (EHR). While the observer recorded that epinephrine was given within 5 min of arrest 90% of the time, EHR documentation reported an 81% occurrence (kappa = 0.27, 95 % CI 0.16–0.36).^16^ EMS patient care report narratives may be subject to similar discrepancies in real-time vs. post-event reporting accuracy.

This study has several limitations. We excluded 8.2% (71/868) of available cardiac arrest files due to lack of bystander CPR documentation. The missing audio file data may be due to various reasons: the arrest being called in by police, the call being taken by another public safety answering point, or the caller not being at the scene of arrest. This degree of incomplete data is a constraint among registries. In a national EMS database of 844,021 OHCAs, bystander CPR was missing in 17.9% of patient care reports.^17^ Nonetheless, missingness may reduce statistical power and introduce bias. Given the observational nature, there is potential for recall bias as medics may not complete their documentation until after they hand-off care to the receiving facility. Since audio review relies solely on auditory cues and lacks direct visual confirmation, it may overestimate bystander CPR provision. However, the emerging use of video-assisted telecommunicator-instructed CPR may improve the accuracy of this in the future.^18^ In 2016, medical directors began to routinely review telecommunicator operator recordings and files for quality assurance, potentially resulting in performance bias among this group.

One step systems could take to address these discrepancies is to employ automatic sharing of dispatch CPR data into patient care report systems. By populating patient care report fields with real-time dispatch indicators (e.g., confirmed bystander CPR), systems can reduce missing documentation and prompt EMS clinicians to verify or reconcile discrepancies early. Such automation is achievable with existing computer aided dispatch‑patient care report linkages and requires minimal workflow change while offering high returns in data quality and research fidelity.

Systems could also employ an expanding array of machine learning tools to screen for data entry errors either retrospectively or in real-time. By training machine learning tools on larger, validated clinical data sets, these tools can learn patterns representative of accurate entries and thus identify anomalies indicative of errors. Choi et al demonstrated the efficacy of this approach in their use of a machine learning tool trained with a national cardiac arrest registry to analyze prehospital patient care reports*.*^19^

## Conclusion

In this single-center, urban EMS agency, we found moderate agreement in the documentation of bystander CPR between emergency call dispatch file review and EMS clinician documentation. A higher incidence of bystander CPR was measured when reviewing dispatch audio files. These findings highlight potential inconsistencies in how bystander CPR is identified and documented across the emergency response system and underscore the importance of standardized reporting practices to ensure accurate data for quality improvement and research.

## Declaration of generative AI and AI-assisted technologies in the writing process

During the preparation of this work the author(s) used Gemini, a large language model from Google**,** to improve the language and readability. After using this tool/service, the author(s) reviewed and edited the content as needed and take(s) full responsibility for the content of the publication.

## Acknowledgements/Funding

This study was supported by the University of Utah’s REDCap infrastructure, funded by the National Center for Advancing Translational Sciences of the 10.13039/100000002National Institutes of Health [UL1TR002538].

Helen Palatinus is supported by the National Institutes of Health [5U24NS100657-08, sub-award No. 1021802, via 10.13039/100006668Oregon Health & Science University].

## CRediT authorship contribution statement

**Helen N. Palatinus:** Writing – review & editing, Writing – original draft, Methodology, Investigation, Formal analysis, Data curation, Conceptualization. **Ashlynn A. Felker:** Writing – review & editing, Writing – original draft, Methodology, Investigation, Data curation. **Tate Colton:** Writing – original draft, Data curation. **Graham Brant-Zawadzki:** Writing – review & editing, Writing – original draft. **Scott T. Youngquist:** Writing – review & editing, Writing – original draft, Supervision, Methodology, Investigation, Formal analysis, Data curation, Conceptualization.

## Declaration of competing interest

The authors declare that they have no known competing financial interests or personal relationships that could have appeared to influence the work reported in this paper.
